# A Time-Resolved Fluorescent Microsphere Immunochromatographic Assay for Determination of Vitamin B12 in Infant Formula Milk Powder

**DOI:** 10.3390/bios15020065

**Published:** 2025-01-21

**Authors:** Qianqian Lu, Yongwei Feng, Qi Zhou, Ting Yang, Hua Kuang, Chuanlai Xu, Lingling Guo

**Affiliations:** 1 Wuxi Food Safety Inspection and Test Center, Wuxi 214142, China; 7210112038@stu.jiangnan.edu.cn (Q.L.); fyw18961779229@wxifc.org.cn (Y.F.); zq13222808453@wxifc.org.cn (Q.Z.); yt18952479769@wxifc.org.cn (T.Y.); 2 Technology Innovation Center of Special Food for State Market Regulation, 35-302 South Changjiang Road, Wuxi 214142, China; 3 International Joint Research Laboratory for Biointerface and Biodetection, School of Food Science and Technology, Jiangnan University, Wuxi 214122, China; kuangh@jiangnan.edu.cn (H.K.); xcl@jiangnan.edu.cn (C.X.); 4 State Key Laboratory of Food Science and Resources, Jiangnan University, Wuxi 214122, China

**Keywords:** vitamin B12, monoclonal antibody, immunochromatographic assay, infant formula milk powder

## Abstract

Vitamin B12 (VB12) is an important nutrient, and its quality control in food is crucial. In this study, based on the principle of specific recognition of target analyte by monoclonal antibodies (mAbs), a time-resolved fluorescent microsphere immunochromatographic assay (TRFM-ICA) was developed to detect the content of VB12 in infant formula milk powder. First, the performance of the anti-VB12 mAb was evaluated, revealing a half-maximal inhibitory concentration of 0.370 ng/mL, an affinity constant of 2.604 × 10^9^ L/mol and no cross-reactivity with other vitamins. Then, a highly sensitive TRFM-ICA was developed, with a visual limit of detection of 10 μg/kg and a cut-off value of 100 μg/kg for qualitative detection and a detection range of 4.125–82.397 μg/kg for quantitative detection. In addition, the test results of real samples were consistent with the results of quantification using microbiological methods, with a coefficient of variation of less than 10%, showing good accuracy and stability, and confirming that the TRFM-ICA is suitable for the analysis of VB12 in real infant formula milk powder samples. In this study, based on the principle of specific recognition of VB12 by monoclonal antibodies (mAbs) against VB12, a time-resolved fluorescence microsphere immunochromatographic assay (TRFM-ICA) was developed to detect the content of VB12 in infant formula by converting biological signals into optical signals.

## 1. Introduction

Infant formula milk powder is a special dietary food that is of great significance for the growth and development of infants [1,2]. However, there are still many food safety issues with infant formula milk powder, and the types and contents of nutrients vary among different products [3]. Vitamin B_12_ (VB_12_), also known as cobalamin, is a general term for a class of corrinoid compounds containing cobalt [4]. It is the only water-soluble vitamin that contains a metal ion and is essential for the human body, and it is also an important indicator of the nutritional value of infant formula milk powder [5,6]. VB_12_ mainly participates in the regulation of epigenetic mechanisms in the human body, regulating the normal metabolism of carbohydrates, proteins, and fats, maintaining the function of the nervous system, and preventing mental diseases such as Alzheimer’s disease and depression [7,8]. VB_12_ deficiency can cause anemia, hyperhomocysteinemia, and neuropsychiatric diseases, as well as fertility problems and birth defects [9], and infants often show growth retardation and nervous system defects [10]. However, excessive VB_12_ can also exert toxic side effects on the body. Studies have shown that excessive intake of VB_12_ causes obvious allergic symptoms such as urticaria, eczema, and edema, as well as symptoms such as persistent nerve excitement and palpitations [11]. In China, it is stipulated that the content of VB_12_ added to infant formula should be in the range of 0.5 μg/100 g–7.2 μg/100 g [12]. Consequently, accurate determination of VB_12_ content in infant formula milk powder is crucial.

The determination of VB_12_ has gradually improved, with established methods including spectroscopy [13,14], electrochemical methods [15], chromatography [16], and chromatography–mass spectrometry [12,17,18]. However, these methods are expensive and require time-consuming pretreatment steps and complex equipment [19,20]. In addition, immunoassays have also been used for the detection of VB_12_, mainly including ELISA [21,22] and immunochromatographic assay [23,24]. Among them, ELISA operation is relatively complex and not suitable for rapid detection of a large number of samples, while immunochromatographic assays have received widespread attention due to their potential for simplicity, automation, and low cost, and have great application value. The principle of immunochromatographic assays is based on the specific recognition of antigens by high-performance antibodies, converting biological signals into light signals for qualitative or quantitative analysis [25]. Multiple signal markers have been used to amplify reaction signals and enhance the sensitivity of detection, including enzyme markers such as alkaline phosphatase [26] and fluorescent or chemical labeling materials such as fluorescent microspheres [27] and metal-organic frameworks [28]. Some redox systems have also been used to amplify detection signals, such as electrochemical immunosensors involving redox cycling amplification [29]. Additionally, dual-signal immunosensors have further improved signal sensitivity and accuracy due to their advantages of wide detection ranges and high detection sensitivity [30].

Among these signal amplification methods, time-resolved fluorescent microspheres (TRFMs) combine lanthanide element labeling as a tracer chelate with fluorescence measurement, with long half-life and long stoke shift, which are conducive to reducing the interference of non-specific fluorescence signals [31]. Concurrently, the surface of the fluorescent microspheres is modified with an optimal density of carboxyl functionalities to facilitate covalent bonding with antibodies, improving the stability of the labeling material. In practical detection, the combination of time-resolved fluorescent microspheres with lateral flow assays can shorten the detection time and simplify the detection operations. It has already been used for the detection of biotoxins, antibiotics, and prohibited additives [32]. However, time-resolved fluorescence immunoassay technology still has considerable room for development, with specificity, accuracy, and sensitivity all capable of continuous improvement to meet diverse detection needs.

Therefore, in this study, a time-resolved fluorescent microsphere immunochromatographic assay (TRFM-ICA) was developed for VB_12_ determination in infant formula milk powder, using time-resolved fluorescent microspheres as the labeling material and based on a high-performance anti-VB_12_ monoclonal antibody (mAb). This method is characterized by rapidity, high sensitivity, and good stability, and suitable for on-site monitoring of a large number of samples.

## 2. Materials and Methods

### 2.1. Equipment and Chemicals

Vitamin standard analytes, Freund’s adjuvant, horseradish peroxidase-labeled goat anti-mouse immunoglobulin (IgG), and 3,3,5,5-tetramethylbenzidine were purchased from J&K Scientific (Beijing, China). Negative and positive infant formula milk powder were collected from the Food Safety Inspection and Testing Center (Wuxi, China). The components of TRFM-ICA were obtained from Jieyi Biotechnology Co., Ltd. (Shanghai, China). The VB_12_ coating antigen and anti-VB_12_ mAbs were developed in our laboratory. TRFMs were purchased from NanoGen Biotechnology Co., Ltd. (Beijing, China). A time-resolved immune analyzer (FIC-H1) was obtained from Determine Biotechnology Co., Ltd. (Wuxi, China).

### 2.2. Production of Anti-VB_12_ mAb

The mAbs were prepared according to previous studies [33], including mice immunization, cell fusion, ascites preparation and purification. The immunogen was synthesized by conjugating VB_12_ and BSA using the mild acid hydrolysis method [22]. The initial immunization dose was 100 μg, and the booster immunization dose was 50 μg. After five immunizations, ic-ELISA was used to select the best performing mouse for cell fusion. Hybridoma cells producing anti-VB_12_ antibodies were generated by fusing mouse spleen cells with SP 2/0 myeloma cells. The best performing cell lines were selected through cell subcloning, expanded, and injected into the abdominal cavity of mice to produce ascites. And the ascites was purified using caprylic–acid–ammonium sulfate precipitation to obtain anti-VB12 mAbs.

### 2.3. Property Determination of Anti-VB_12_ mAb

High-performance mAbs contribute to the establishment of immunoassay methods, and the subtype, affinity, sensitivity, and specificity of the anti-VB_12_ mAb were analyzed by ELISA [34]. The subtypes were determined using an isotyping kit, and the result was determined by color development.

Antibody affinity was determined by direct ELISA and represented by the affinity constant (Ka). The mAb was diluted to different concentration gradients and measured at different coating antigen concentrations. Standard curves were established with absorbance values as the vertical coordinate and mAb concentrations as the horizontal coordinate. The calculation formula for Ka was as follows:

Ka = (n − 1)/2 (n [Ab′]_t_ − [Ab]_t_)

where [Ab′]_t_ and [Ab]_t_ refer to the corresponding molar concentrations of mAbs at OD_max_/2 for two coating concentrations, and n refers to the ratio of the two coating concentrations (n > 1).

Antibody sensitivity was determined by ic-ELISA, and pH and NaCl concentrations in the buffer were optimized. Under optimal conditions, a series of VB_12_ standards were measured, and a standard curve was fitted. The sensitivity of the anti-VB_12_ mAb was represented by the half-maximal inhibitory concentration (IC_50_) of the standard curve.

Moreover, the specificity of the mAb was also evaluated by ic-ELISA. The specificity was evaluated based on the cross-reactivity (CR) ascertained by measuring the IC_50_ values of a series of vitamins. The calculation formula for CR was as follows:

CR (%) = (IC_50_ of VB_12_/IC_50_ of other vitamins) × 100%.

### 2.4. Production of Fluorescent Microsphere-Labeled Monoclonal Antibody

Fluorescent nanospheres and mAbs were coupled through an amidation reaction between the carboxyl groups on the microsphere surface and the amino groups of bioproteins using the active ester method [35]. First, 100 μL of carboxyl-modified fluorescent microspheres were added to 100 μL of MES solution (pH 6.0, 0.02 M) and vortexed for 2 min to evenly disperse the fluorescent microspheres. Then, 20 μL of 1-ethyl-3-(3-dimethylaminopropyl) carbodiimide (EDC) solution (4 mg/mL) and 20 μL of N-hydroxysuccinimide (NHS) solution (4 mg/mL) were added, and the mixture was stirred at room temperature for 20 min. Subsequently, the mixture was centrifuged at 11,000× *g* for 15 min and resuspended in carbonate buffer (pH 9.5, 0.05 M); then, 50 μL of anti-VB_12_ mAb (0.2 mg/mL) was added, and the mixture was reacted at room temperature for 2 h. Next, 100 μL of a solution containing 10% BSA was added and reacted for 2 h. The mixture was then centrifuged at 11,000× *g* for 15 min, and the precipitate was resuspended in a resuspension solution containing 5% sucrose, 0.5% BSA, and 1% Tween-20 to obtain a solution of fluorescent microsphere-labeled monoclonal antibody (TRFM-mAb). The TRFM-mAb solution was diluted with a diluent to an appropriate multiple, sprayed onto a glass fiber pad, and dried in a 37 °C blast drying oven for 12 h to form the conjugate pad.

### 2.5. Assembly of the Fluorescent Immunochromatographic Test Strip

**Figure 1:**
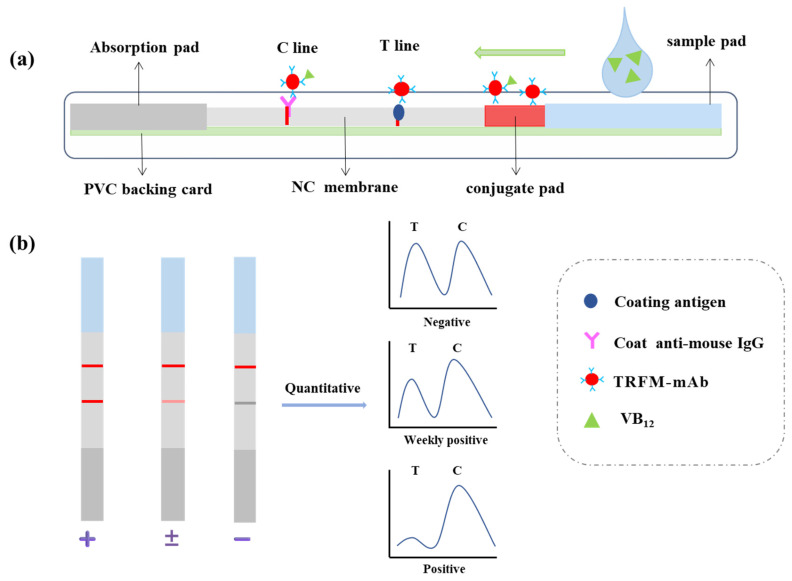
Principles (**a**) and test results (**b**) of TRFM-ICA.

The structure of the immunochromatographic test strip is shown in Figure 1a. First, the NC membrane was pasted in the central area of the PVC base plate, and the coating antigen and goat anti-mouse IgG antibody were fixed in the middle area of the NC membrane to form the test line (T line) and the control line (C line), respectively, with an interval of 4 mm. Then, the baseplate was dried in a 37 °C blast drying oven for 12 h to form the detection area of the immunochromatographic test strip. The absorbent pad and the conjugate pad were fixed on either side of the PVC base plate, with 2 mm overlapping with the NC membrane. Next, the sample pad was attached to the PVC base plate. Finally, the base plate was cut into strips and loaded into a card shell.

### 2.6. Sensitivity Determination of TRFM-ICA

To achieve optimal sensitivity, we optimized the concentration of the T line antigen, the type of surfactant in the TRFM-mAb resuspension solution, and the optimal dilution factor of the microspheres. Under optimal conditions, six standard solutions with different concentrations of VB_12_ were measured, and a corresponding standard curve was fitted to determine the sensitivity of TRFM-ICA.

### 2.7. Sample Analysis

One gram of blank milk powder was weighed and spiked with VB_12_ at concentrations of 0, 5, 10, 20, 50, and 100 μg/kg. Then, 3 mL of 55 °C warm water was added and shaken to mix evenly. The mixture was ultrasonicated at 60 °C for 30 min, centrifuged at 15,000× *g* for 15 min. The supernatant was then diluted to an appropriate multiple with PBS containing 1% ON-870, and 100 μL of the solution was taken for sample testing.

To evaluate the stability and accuracy of the test strip, positive samples quantified by microbiological methods were tested using the TRFM-ICA, and the results were analyzed and compared.

## 3. Results and Discussion

### 3.1. Principle of the TRFM-ICA

Qualitative and quantitative analysis of the target analyte was achieved by TRFM-ICA based on the principle of competitive inhibition immunochromatography [36]. As shown in Figure 1a, the sample solution flowed along the conjugate pad and NC membrane towards the absorbent pad under capillary action. If VB_12_ was present in the sample, the TRFM-mAb first binded to VB_12_ in the sample, thereby inhibiting the binding of TRFM-mAb to the coating antigen on the T line. The higher the content of VB_12_ in the sample, the less TRFM-mAb bound to the T line, resulting in a decrease in the fluorescence intensity of the T line. The goat anti-mouse IgG antibody on the C line captured both TRFM-mAb that did not bind to the target analyte and TRFM-mAb that bound to the target analyte. Therefore, regardless of whether the sample contained VB_12_, the C line always showed a signal. The measurement results are shown in Figure 1b. For qualitative analysis, visual limit of detection (vLOD) and cut-off values were introduced and defined as the VB_12_ concentrations corresponding to the T line beginning to lighten and completely disappearing, respectively. For quantitative analysis, the fluorescence intensity was read using a time-resolved immune analyzer. A standard curve was fitted with the T/C value as the vertical coordinate and the concentration of VB_12_ as the horizontal coordinate and the corresponding concentration was measured by importing the curve into the analyzer. The calculated limit of detection (cLOD) represented the sensitivity of quantitative detection, calculated as the VB_12_ concentration corresponding to y_(x=0)_ − 3SD.

### 3.2. Evaluation of mAb Performance

**Figure 2:**
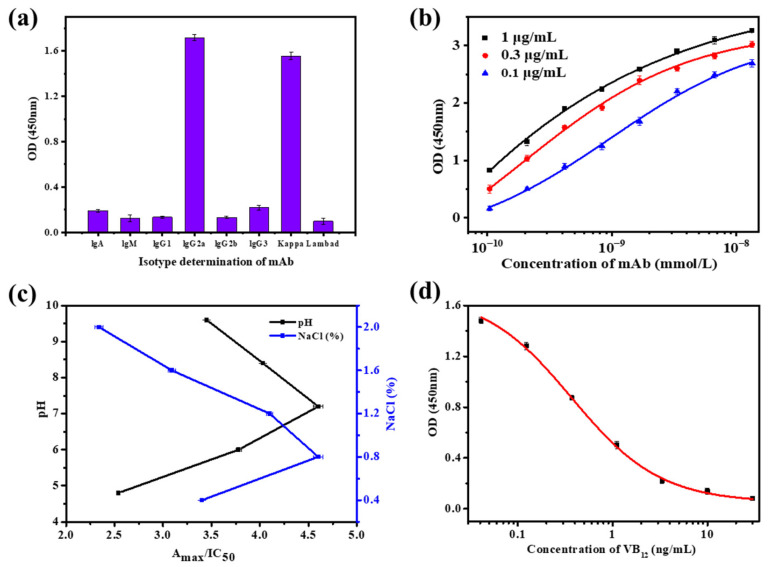
Performance testing of the anti-VB_12_ mAb. (**a**) Isotype measurement. (**b**) Affinity measurement. (**c**) Sensitivity optimization. (**d**) Sensitivity measurement.

Antibodies are glycoproteins derived from the immune system and exist as one or more “Y” monomers. Their structure consists of two pairs of identical heavy chains and light chains. As shown in Figure 2a, the subtypes of anti-VB_12_ mAb were IgG_2a_ and Kappa, with high purity.

The mAbs with high affinity can bind more antigens in shorter time. As shown in Figure 2b, the affinity constant (Ka) of the anti-VB_12_ mAb was 2.604 × 10^9^ L/mol; thus, it had a high affinity, and it is suitable for the establishment of the immunoassay method.

As an important indicator for evaluating antibody quality, antibody sensitivity is of great significance for the practical application of mAbs. As shown in Figure 2c, when the pH was 7.2 and the NaCl concentration was 0.8%, Amax/IC_50_ was maximized, and anti-VB_12_ mAb exhibited the highest sensitivity. Under these conditions, the IC_50_ of VB_12_ was 0.370 ng/mL, meeting the sensitivity requirements of TRFM-ICA detection. The specificity results of the mAbs are shown in Table 1, and anti-VB_12_ mAb showed no cross-reactivity with other vitamins, indicating a highly specific antibody which does not produce false positives during the establishment of TRFM-ICA.

### 3.3. Sensitivity of the TRFM-ICA

**Figure 3:**
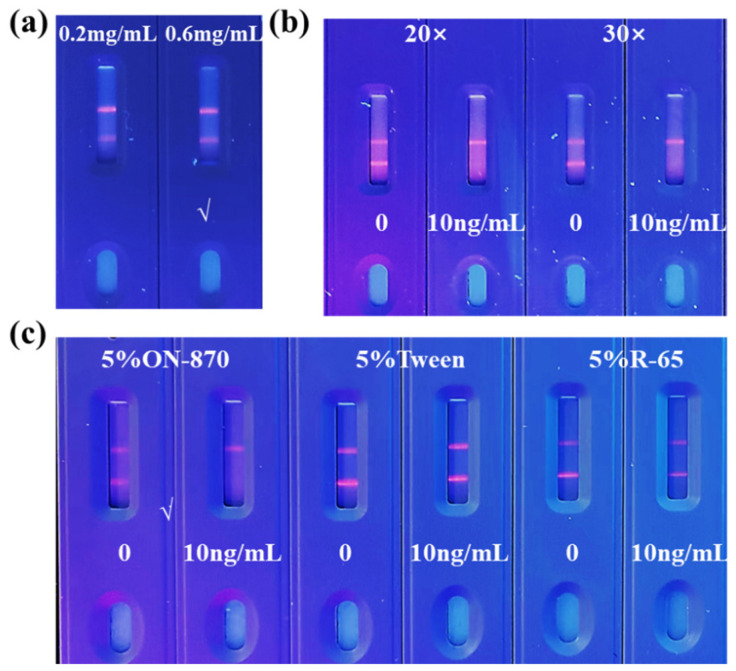
Optimization of TRFM-ICA. (**a**) Optimization of coating antigen concentrations in T line. (**b**) Optimization of dilution ratio of microspheres. (**c**) Optimization of surfactant.

**Figure 4:**
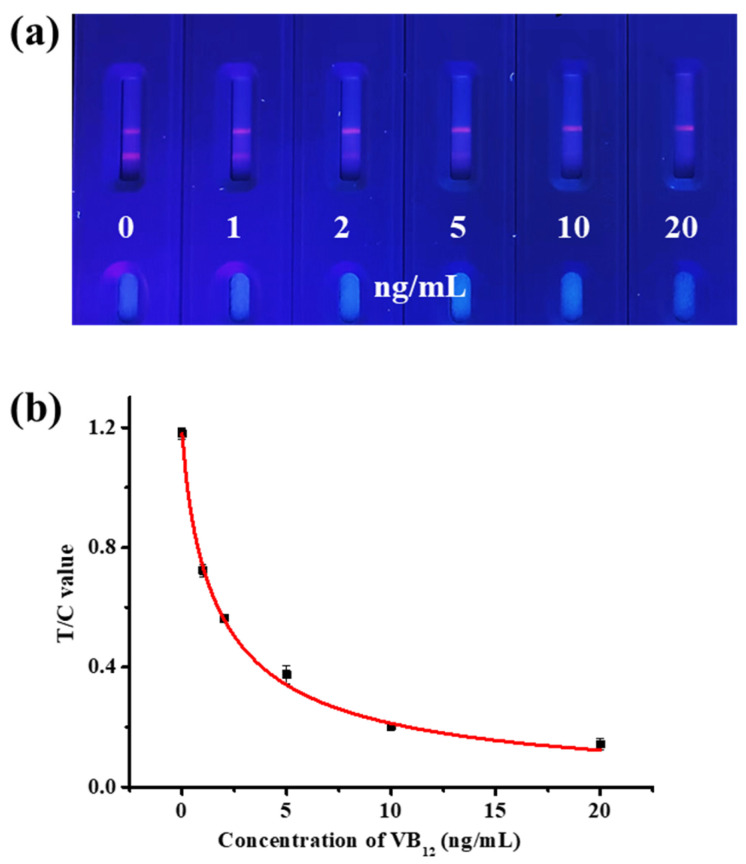
Sensitivity measurement of TRFM-ICA. (**a**) Images of sensitivity testing. (**b**) Standard curve of VB_12_ detection.

Appropriate antigen concentration and microsphere dilution factors contribute to the color development of the T line and improving the sensitivity of TRFM-ICA. As shown in Figure 3a,b, when the antigen concentration on the T line was 0.6 mg/mL, the T line showed clear fluorescence intensity at 0 ng/mL VB_12_. For the microsphere dilution factor, when the microspheres were diluted 30 times, the T line showed higher fluorescence intensity at 0 ng/mL VB_12_, and the T line was completely invisible at 10 ng/mL VB_12_, showing good sensitivity. When the microspheres were diluted 20 times, the concentration was too high, resulting in unclean chromatography. In addition, due to the larger particle size of the fluorescent nanospheres, the use of surfactants was needed to increase their mobility on the chromatographic test strip. Different substances have different effects on flow rate and antibody performance [37]. As shown in Figure 3c, when the surfactant was 5% ON-870, the T line showed clear fluorescence intensity at 0 ng/mL VB_12_, and the T line was completely invisible at 10 ng/mL VB_12_. Therefore, 5% ON-870 was chosen to determine the sensitivity of TRFM-ICA. As shown in Figure 4a, a series of VB_12_ contents in PBS were measured under optimal conditions and produced vLOD and cut-off values of 1 ng/mL and 10 ng/mL, respectively. Figure 4b shows the quantitative standard curve for VB_12_ determination in PBS, with the curve equation of y = −0.012 + 1.191/(1 + [x/1.823]^0.857^) and a cLOD of 0.059 ng/mL, showing high sensitivity and indicating that the TRFM-ICA is suitable for the detection of real samples.

### 3.4. Application of TRFM-ICA

**Figure 5:**
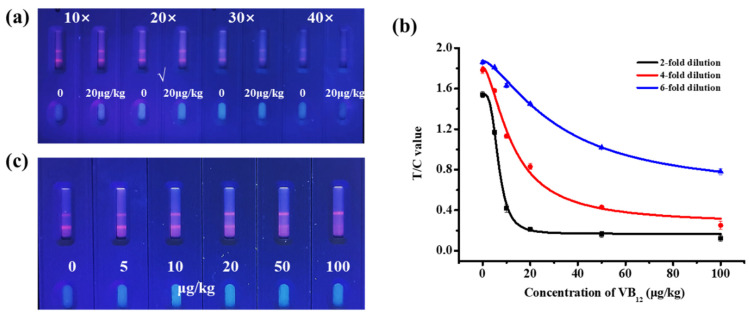
Test results of TRFM-ICA in infant formula milk powder. (**a**) Optimization of dilution ratio of microspheres. (**b**) Standard curves for different dilution ratios of sample extraction solutions. (**c**) Image of measurement result of 6-fold dilution of sample extraction solution.

To determine the anti-matrix interference ability of TRFM-ICA, infant formula milk powder was analyzed using TRFM-ICA. First, the dilution factor of the microspheres was optimized. From Figure 5a, when the fluorescent microspheres were diluted 20 times, the T line showed strong fluorescence intensity at 0 μg/kg VB_12_, and the T line became significantly lighter at 20 μg/kg VB_12_. Therefore, a 20-fold dilution was chosen to evaluate the real samples. A series of VB_12_ concentrations were spiked into the infant formula milk powder and assayed using TRFM-ICA. Here, the dilution factor of the sample extract was optimized to minimize matrix interference and select an appropriate detection range. As shown in Figure 5b, two-fold and four-fold dilutions resulted in higher sensitivity, but their detection ranges were narrow and not suitable for the analysis of VB_12_ in different infant formula milk powders. The detection range of the six-fold dilution was 4.125–82.397 μg/kg, which was basically able to meet the determination of VB_12_ in different infant formula milk powders. Therefore, a six-fold dilution was chosen as the dilution factor for the sample extract. Figure 5c shows the qualitative results obtained with infant formula milk powders, with vLOD and cut-off values of 10 μg/kg and 100 μg/kg, respectively. Therefore, TRFM-ICA was suitable for VB_12_ detection in infant formula milk powder, with a wide range of applications, high sensitivity, and rapid detection ability.

### 3.5. Analysis of VB_12_ in Infant Formula Milk Powder

To determine the accuracy and stability of TRFM-ICA, positive samples quantified by microbiological methods were analyzed using TRFM-ICA. As shown in Table 2, results of VB_12_ measurement by TRFM-ICA showed good consistency with positive samples, with a relative recovery rate of 84.0–111.7% and a coefficient of variation (CV) value less than 10%. And stability experiments were conducted, storing the test strips in a sealed and dry environment at 25 °C for 180 days. The measurement results of Samples 3 and 4 remained consistent with the microbiological method measurement results (Table 3). Therefore, this method exhibited good accuracy and stability, and the measurement results were reliable. In addition, compared with other immunoassay methods, TRFM-ICA has the advantages of high sensitivity and fast detection speed (Table 4), showing great development prospects for the analysis of VB_12_ in infant formula milk powder.

## 4. Conclusions

In this study, the performance of the anti-VB_12_ mAb was first evaluated. The mAb had an IC_50_ of 0.370 ng/mL, an affinity constant of 2.604 × 10^9^ L/mol, and no cross-reactivity with other vitamins, showing high sensitivity, affinity, and specificity. Based on mAb, a TRFM-ICA for rapid detection of VB_12_ in infant formula milk powder was established. This method can be used for both qualitative and quantitative detection. For qualitative detection, the vLOD value is 10 μg/kg, and the cut-off value is 100 μg/kg. For quantitative detection, the detection range is 4.125–82.397 μg/kg. The results of real sample measurements showed that this method has a relative recovery rate of 84.0–111.7% compared to the microbiological method, with a CV of less than 10%, making it suitable for on-site rapid detection of VB_12_ in infant formula milk powder. This method requires simple pretreatment and provides a rapid detection process, showing a wide range of development opportunities for VB_12_ detection.

## Figures and Tables

**Table 1:** Cross-reactivity of anti-VB_12_ mAbs with other vitamins.

Vitamins	IC_50_ (ng/mL)	CR (%)
VB_12_	0.370	100
Vitamin B_1_	>100	<1
Vitamin B_2_	>100	<1
Vitamin B_3_	>100	<1
Vitamin B_5_	>100	<1
Vitamin B_6_	>100	<1
Vitamin B_7_	>100	<1
Vitamin B_9_	>100	<1

**Table 2:** Real samples analysis using TRFM-ICA.

Samples	The Determination Value of Microbiological Method (μg/kg)	TRFM-ICA		Relative Accuracy (%) ± SD
		Detection Value (μg/kg) Mean ± SD	CV (%)	
1	68.09	60.23 ± 1.35	2.2	88.4 ± 2.0
2	64.02	69.37 ± 2.88	4.2	108.4 ± 4.5
3	30.30	32.45 ± 1.09	3.4	107.1 ± 3.6
4	72.21	60.67 ± 3.03	5.0	84.0 ± 4.2
5	30.03	26.29 ± 0.67	2.5	87.6 ± 2.2
6	32.11	30.35 ± 1.98	6.2	94.5 ± 6.2
7	37.28	41.67 ± 1.43	3.4	111.7 ± 3.8
8	40.80	35.29 ± 2.32	6.6	86.5 ± 5.7
9	34.81	30.37 ± 0.74	2.4	87.3 ± 2.1

**Table 3:** Stability test of TRFM-ICA.

Samples	The Determination Value of Microbiological Method (μg/kg)	Test Results After 180 Days by TRFM-ICA		Relative Accuracy (%) ± SD
		Detection Value (μg/kg) Mean ± SD	CV (%)	
3	30.30	32.19 ± 1.27	4.0	106.2 ± 4.2
4	72.21	65.32 ± 3.32	5.1	89.8 ± 4.6

**Table 4:** TRFM-ICA comparison with other immunoassay.

Method	Samples	LOD (ng/mL)	Measuring Time
TRFM-ICA	Infant formula milk powder	0.059	15 min
ELISA [21]	Seawater	0.2	80 min
ELISA [22]	Vitamin injections, tablets capsules and chocolates	10	90 min
Immunodipstick-based gold nanosensor [23]	Fruit and energy drinks	1	/
Dipstick-based immunochemiluminescence biosensor [24]	Energy drinks	1	10 min
Competitive chemiluminescent enzyme immunoassay [38]	Milk	0.08 ng/mL	97 min
ELISA [39]	Vitamin tablets, energy drink, and infant milk powder	0.065	75 min
ELISA [40]	VB_12_ tablet supplements	0.2	133 min

## Data Availability

The raw data supporting the conclusions of this article will be made available by the authors on request.
